# Support groups for caregivers of patients with Dementia: A comparative study

**DOI:** 10.1590/s1980-57642008dn10200013

**Published:** 2007

**Authors:** Maria Paula Foss, Celmira Lange, José Humberto Silva Filho, Fabiana Brunini, Francisco A. Carvalho do Vale

**Affiliations:** 1 Post-graduate student. Faculty of Medicine of Ribeirão Preto, University of São Paulo; 2 Nurse, PhD. Faculty of Medicine of Ribeirão Preto, University of São Paulo; 3 Post-graduate student. Faculty of Philosophy, Science and Language of Ribeirão Preto, University of São Paulo; 4 Social Work. Faculty of Medicine of Ribeirão Preto, University of São Paulo; 5 MD, PhD, Neurologist. Faculty of Medicine of Ribeirão Preto, University of São Paulo.

**Keywords:** caregivers, dementia and non-medicamentous interventions

## Abstract

Since the dementias lead to the need for caregivers, a Group for Dementia Caregivers (GC) was set up where the present study compared the psychosocial characteristics, quality of life and caregiver overload in a Group For Dementia Caregivers (GC) with caregivers not involved in this scheme (non–GC).

**Methods:**

19 primary caregivers counseled by this group (GC group) was studied and compared to a group of 13 caregivers not receiving such counseling (non-GC group). The instruments used were the World Health Organization Quality of Life (WHOQOL-bref), the State-Trait Anxiety Inventory (STAI) and Caregiver Load Scale (CLS).

**Results:**

The two groups did not differ in mean age or gender (P<0.05). GC caregivers had a higher educational level, were service workers where majority were the children of patients. In the Non-CG group, the most frequent occupation was housewife, with most subjects being spouses. The WHOQOL revealed a significant difference (p<0.05) between groups in the physical, social relations and environment domains (GC>Non-GC). The STAI revealed a significant difference (p<0.05) in the Trait subscale (GC>Non-GC), but not in the State subscale. There was no significant difference in CLS.

**Conclusion:**

The GC appeared to be of benefit to its participants, with probable positive repercussions on the patients, particularly regarding their quality of life.

## Abstract (pt)

Desde que as demências levam a necessidade de cuidadores, um Grupo para Cuidadores de Pessoas com Demência (GC) formou-se e no presente estudo comparou-se as variáveis psicossociais, qualidade de vida, ansiedade e sobrecarga do cuidador num Grupo para Cuidadores de Pessoas com demência (GC) e outro com cuidadores que não estavam envolvidos nesse serviço (não-GC).

**Métodos:**

19 cuidadores primários participantes do grupo (grupo GC) foram estudados e comparados com 13 que não participaram desses grupos (grupo não-GC) Os instrumentos utilizados foram Qualidade de Vida da Organização Mundial de Saúde (WHOQOL-abreviada), Inventário de Ansiedade Traço-Estado (IDATE) e Escala de Carga do Cuidador (ECC).

**Resultados:**

Os dois grupos não diferiram em relação à média de idade e o gênero (p<0,05). Os cuidadores do GC tinham maior escolaridade e eram trabalhadores de serviços, sendo a maioria deles filhos dos pacientes. No Não-GC a ocupação mais freqüente foi de donas de casa, sendo a maioria cônjuges. Na WHOQOL, houve diferença significativa (p<0,05) entre os grupos, nos domínios físico, relações sociais e meio ambiente (GC>não GC). No IDATE, houve diferença significativa (p<0,05) na subescala Traço (GC>não GC), mas não na subescala Estado. Não houve diferença significativa na ECC.

**Conclusão:**

O GC pareceu beneficiar seus participantes, com prováveis repercussões positivas sobre os pacientes, principalmente na qualidade de vida dessas pessoas.

The increased longevity of the population has led to a higher incidence of mental disorders, including the dementias. The dementia syndromes are clinical pictures usually directly associated with aging, and primarily characterized by memory impairment combined with one or more clinical conditions such as aphasia, apraxia, agnosia and perturbations of executive functioning that damage the social and occupational functioning of an individual, as stated by the Diagnostic and Statistical Manual of Mental Disorders of the American Psychiatry Association, DSM-IV.^1^

Studies conducted in different parts of the world have demonstrated different prevalence of dementias, although they are all significant, having a progressive characteristic. The prevalence of dementia after 65 years of age is 2.2% in Africa, 5.5% in Asia, 6.4% in North America, 9.4% in South America, and 9.4% in Europe. Specifically in Brazil, the prevalence of dementias is estimated at 7.1%, with 55.1% of the affected patients having Alzheimer Disease.^2,3^

The increased life expectancy for the population, health policies that stimulate deinstitutionalization together with the economic and social rationale of the last decades have promoted a return of the patients to their family context and a corresponding need for caregivers who play a key role in keeping the patient in the community. Caregivers can be divided into primary and secondary types, with primary caregivers assuming the main responsibility for direct assistance to dementia patients (care regarding safety, hygiene, feeding, medication, social mediation etc.), while secondary caregivers help the patients regarding their needs but are not the main persons responsible for them.^4^

The primary caregiver in general will combine these new responsibilities with pre-existing professional, family, social and marital functions, thus being more subjected to overload and stress. Thus, they will be more vulnerable to physical and emotional diseases compared to the rest of the population. Kiecolt-Glaser et al.^5,8^ and Glaser et al.^6,7^ have provided evidence of the mechanisms by which chronic stressors (caregiving for a spouse with progressive dementia) may accelerate the risk of a host of age-related diseases by prematurely aging the immune response. However, although caregivers exhibited higher sympathetic activation than noncaregivers, their magnitude of autonomic and neuroendocrine reactivity was comparable across these groups, suggesting that the effects of chronic stress on physiological reactivity may be less robust in older adults.^9^

Despite recent studies on the quality of life of caregivers of patients with dementia, Matsui et al.^10^ developed a Japanese version of the Quality of Life-Alzheimer’s disease (QOL-AD) because this variable has not yet been fully investigated in subjects with impaired cognitive ability due to dementia. Taub, Andreoli and Bertolucci^11^ also developed a Brazilian version of the Zarit Caregiver Burden Interview, with sufficient reliability, proving comparable to the original version. Both of these studies occurred after the current work, therefore we adopted the World Health Organization Quality of Life (WHOQOL-bref) instrument which was both available and adapted to Portuguese. The WHOQL according to the World Health Organization defines quality of life as the perception by an individual of physical, mental and social well-being with respect to their objectives, expectations, standards and concerns.^17^ For this reason, it is recommendable that the health professionals involved in the care of persons with dementia provide, in addition to the treatment of the patient, multidisciplinary psychosocial care for patients’ relatives and caregivers. Lopes and Bottino^2^ demonstrated that interventions with relatives and caregivers reduced the psychiatric symptoms they experienced, significantly improving the well-being of patients and caregivers alike.

Others suggest that including a family-system component in caregiver interventions may be beneficial in reducing caregiver burden in these very distressed individuals.^12^ Caregivers’ distress can also be studied in terms of behavioral symptoms. Vugt et al.^13^ emphasized the importance of differentiating between diagnostic groups and specific behavioral domains when focusing on caregiver reactions to problem behavior. These ideas could prove important in developing future studies in the area.

Also, the assessment of the efficacy of group interventions is complicated by all the variables involved. Thus, Rabinowitz^14^ in his work, advocates the use of self- efficacy as a screening tool for appropriate caregiver intervention assignment, a factor that could also contribute in futures studies.

The present study was conducted in the Behavioral Neurology Outpatient Clinic (ANCP in the Portuguese acronym) of the University Hospital of the Faculty of Medicine of Ribeirão Preto (HCFMRP) which is a multidisciplinary entity that has attended more than 1,400 patients to date, more than half of whom are persons with dementia who live with their family and have their spouse or one of their children as a caregiver. In view of this, the ANCP organized a multiprofessional service aimed at caregivers of persons with dementia, called Groups For Dementia Caregivers (GC) and devised the present study to compare the psychosocial characteristics, quality of life and caregiver overload in a Group For Dementia Caregivers (GC) with caregivers not involved in this service (non–GC).

## Methods

The study was approved by the Research Ethics Committee of the Institution and was specifically conducted on the clientele of ANCP, University Hospital of the Faculty of Medicine of Ribeirão Preto, University of São Paulo, from May to June 2003.

### Casuistic

The participants in the study were primary caregivers whose patients had received a diagnosis of dementia at the ANCP outpatient unit. They were divided into two groups, one of which received intervention (GC, 79% women and 21% men) while the other was used as a control (Non-GC, 92% women and 7.7% men). The GC consisted of 19 caregivers who voluntarily agreed to adhere to the guidance service and who had participated in at least three sessions of the Group for Caregivers. The Non-GC, used as a control, consisted of 13 caregivers who, although having been invited, did not participate in the guidance sessions offered by the group.

### Instruments

We adopted the WHO quality of life scale, short version (WHOQOL-bref)^17^, which contains 26 items and evaluates how a subject feels about their quality of life, health and other areas, considering their values, pleasures and concerns. The scale was constructed according to the health framework adopted by the WHO and is based on a wide gamut of aspects included in the following domains: physical (pain and discomfort, energy and fatigue, sleep and rest, mobility, daily life activities, dependence on medication and treatment, and ability to work); psychological (positive feelings, thinking, learning, memory and concentration, self-esteem, body image and appearance, and negative feelings); social relations (personal relations, social support, sexual activity); environment (physical safety and protection, home environment, financial resources, health and social care, opportunity to acquire new information, participation in recreation and leisure opportunities, and transportation).

The second instrument adopted was the State Trait Anxiety Inventory (STAI)^18^ which contains 40 questions divided into two subscales, i.e., Trait anxiety, and State anxiety. The trait subscale contains 20 statements that require a description of how the person generally feels, characterizing a relatively stable tendency to react to elements considered to be threatening. The state anxiety subscale, also containing 20 statements, investigates the feelings experienced at a given time, referring to a transitory emotional state that varies with time and with the intensity of the triggering stimuli.

Finally, the third instrument adopted was the Caregiver Overload Scale, São Paulo version.^4^ This scale was adapted and validated for the Brazilian culture and consists of 22 questions organized into five dimensions (general tension, isolation, disappointment, emotional involvement, and environment) showing good reproducibility and validity for the measurement of the impact of the disease on the caregivers of patients with chronic diseases (rheumatoid arthritis) and was selected for the present study due to the lack of an instrument with better psychometric qualities. Its objective is to better understand the factors involved in the subjective impact of patient dementia on their caregiver, generating a sensation of burden or overburden as a result of this role.

### Procedures

The GC provided psychoeducational and therapeutic support activities in a cycle of three sessions of approximately 90 minutes each, held every 15 days. The sessions were coordinated jointly by a neuropsychologist, a social assistant and nurses of ANCP, and involved the following topics:

1. Describing the dementia processes with their respective signs, symptoms and orientations.
2. Nursing management and guidance focusing on promoting patient health.
3. Counseling and providing guidance to the caregivers on the importance of the family in giving care, and on the community resources.

The groups were then invited to monthly meetings during which seminars on how to care for oneself and one’s patient were held by the neurologist and other professionals invited, such as lawyers, physiotherapists and others.

All caregivers were invited to participate in the study, and those who agreed signed a Free and Informed Consent Term. In the GC, the questionnaires were applied to each participant during the last session, while in the Non-GC questionnaires were applied to individuals during their regular visits to the ANCP. A semi-structured questionnaire was first applied to obtain data for caregiver identification. Subsequently, the WHOQOL – bref, STAI and Caregiver Load Scale were applied. The instruments were applied by the SGC coordinators, their application being controlled by a single investigator.

### Statistical analysis

The results reported in this paper were based on data collected over the same period, where the following analyses were performed using the SPSS software: the Komogorov-Smirnov test was first used to determine if the numerical variables had a normal distribution, and then the t test was applied to compare the scores for age and education. When the data were not normally distributed, the Mann-Whitney test was applied to the scores in the Caregiver Overload Scale, São Paulo version,^4^ the State Trait Anxiety Inventory (STAI)^18^ and the WHO quality of life scale, short version (WHOQOL-bref).^17^ Gender was analyzed by the Chi-Square Test. The scores for Trait anxiety, and State anxiety were compared by applying the Paired Sample Test. Finally, correlations between all scores were calculated using the Pearson correlation test.

## Results

The psychosocial characteristics of the two groups of caregivers were compared (Table 1). One group consisted of 19 primary caregivers who had participated in GC sessions, while the other consisted of 13 caregivers who had not participated in these sessions (Non-GC). There was no significant difference between groups regarding mean age (in the 50 year range) or gender, with a predominance of women. The white race predominated in both groups, with the classification being based on the caregiver’s self report of skin color.

**Table 1:** Demographic data of the groups studied.

		Group 1	Group 2
Sex	Female	78.9%	92.3%
	Male	21.1%	7.7%
Color	White	63.2%	69.2%
	Black	15.8%	15.4%
	Mulatto	15.8%	15.4%
	Yellow	5.3%	-
Religion	Catholic	78.9%	69.2%
	Evangelical	21.1%	23.1%
	Spiritualist	-	7.7%
Occupation	Active Workers	15.8%	15.4%
	Housewives	52.6%	76.9%
	Retired	15.8%	7.7%
	Unemployed	10.5%	-
	On leave	5.3%	-
Profession	Service Workers	47.4%	15.4%
	Housewives	26.3%	69.2%
	Retired	21.1%	7.7%
	Informal	-	7.7%
Kinship	Spouse	26.3%	56.4%
	Child	52.6%	30.8%
	Sibling	10.5%	7.7%
	Other	10.5%	15.4%
Caregiver	Single	52.6%	76.9%
	Not single	47.4%	23.1%
Types of help	Friends/relatives	15.8%	30.8%
	ANCP	5.3%	-
	None	26.3%	46.2%
	Religion/other	10.5%	7.7%
	More than one	42.1%	15.4%
	type of help		
Age (years)	Mean / SD	51.00/9.55	57.15/13.97
Schooling (years)	Mean / SD	8.16/4.54	4.92/2.99

The caregivers who participated in the GC had a higher educational level, with a predominance of service workers according to the IBGE classification, majority being children of patients. In the Non-GC group the most frequent occupation was that of housewife, with most women being spouses. The caregivers in both groups perceived themselves as the sole persons responsible for the patient even though the participants in the GC mentioned other figures (relatives or social contacts) as aides, whereas Non-GC subjects reported receiving no such help for this task.

Religious practice has often been cited as one of the strategies for coping with stress situations. The Catholic religion predominated in both groups in compared to the Evangelical and Spiritualist religions. However, in the present study the participants were questioned only about the religion they belonged to and not about the practice of this religion. We also did not investigate the greater participation in religious practice by the caregiver in the presence of stress situations, a topic that may be the subject of future studies.

In the evaluation of quality of life (WHOQOL-bref), the caregivers who had participated in the GC demonstrated a significant difference (p≤0.05) in the physical, social relations and environment domains, with higher scores compared to controls (Non-GC). Also on this scale, the psychological domain was the only domain not showing significant differences between the two groups (Table 2).

**Table 2:** Converted scores obtained using the WHOQOL (short form) for participants in the two groups.

Domains	Groups	Mean	Standard deviation	P
Physical	CGG Non-CGG	71.43 55.52	16.96 21.69	<0.05
Psychological	CGG Non-CGG	64.58 59.09	16.18 17.36	0.367
Social relations	CGG	67.54	20.20	<0.05
	Non-CGG	49.31	21.16	
Environment	CGG	61.84	11.25	<0.05
	Non-CGG	49.48	13.58	

In the evaluation of anxiety traits, caregivers who had participated in the GC showed higher levels of anxiety (p≤0.05) compared to Non-GC individuals (Table 3). Regarding the anxiety state, the two groups proved statistically similar (p=0.80).

**Table 3:** Mean percentile obtained with the subscales of the STAI for participants in the two groups.

STAI - Subscales	Groups	Mean percentile	Standard deviation	P
State	CGG	88	9.92	0.80
	Non-CGG	87	12.69	
Trait	CGG	85	14.34	<0.05
	Non-CGG	71	20.58	

Assessment of caregiver overload revealed no significant difference between groups in any of the five dimensions examined (Table 4). The levels of significance appear to demonstrate that the two groups are very similar regarding this overload characteristic involved in the caregiving task. Both groups also had higher total overload scores than the mean values^4^ (global score: mean 1.82; SD 0.59) reported in the literature.

**Table 4:** Scores obtained with the caregiver load scale for participants in the two groups.

	Groups	General tension	Isolation	Disappointment	Emotional involvement	Environment	Total
Mean	CGG Non-CGG	2.13 2.02	1.84 1.75	1.83 1.93	- -	- -	1.95 1.91
Standard deviation	CGG Non-CGG	0.92 0.81	0.89 0.96	0.69 0.78	- -	- -	0.70 0.60
Median	CGG Non-CGG	- -	- -	- -	18.03 14.27	14.13 19.96	- -
P		0.73	0.79	0.70	0.27	0.08	0.87

In order to determine a possible association between the variables evaluated, a correlation study was performed starting with the four domains of the Quality of Life Scale (WHOQOL). An association was detected between the Physical and Psychological domains (correlation coefficient: 0.500, p=0.01) and between the Social Relations and Environment domains (correlation coefficient: 0.771, p=0.01). These data suggest that the internal variables of this scale are directly related to one another in these samples. Similarly, the Caregiver Load Scale revealed correlations between the scores for General Tension and Isolation (correlation coefficient: 0.724, p=0.01), Isolation and Disappointment (correlation coefficient: 0.651, p=0.01); Disappointment and Emotional Involvement (correlation coefficient: 0.482; p=0.01). Again, these variables were directly related to one another. Regarding the anxiety inventory (STAI), there was no correlation between the anxiety trait and the anxiety state.

## Discussion

The GC participants obtained better results in terms of the indicators of quality of life in the physical, social relations and environment domains. The psychological domain was the only domain that did not differ between groups. However, the psychological domain was correlated with the physical, social relations and environment domains, indicating that the latter domains may influence the psychological dimension, causing changes in this sphere. In addition, it should be kept in mind that the dementias are degenerative diseases which cause the caregivers to experience situations of progressive losses, negatively impacting their emotional status. This may partly explain the equivalence of the groups in the psychological domain. On the basis of most of the indicators however, the caregivers participating in the GC seem to demonstrate a better quality of life compared to the Non-GC subjects, a finding that may be associated with the characteristics initially pointed out as distinguishing the two groups. The participation itself of the caregiver in this brief activity of the GC may have been one of the factors contributing to this improvement in quality of life, since among the objectives of the counseling work is access to information about how to care for the patient and how to care for oneself, as well emotional support in view of the burden imposed by this task.

The caregiver, by taking on this greater responsibility in caring for a person with dementia, is more exposed and vulnerable to the state of anxiety, a characteristic seen in both groups. However, it was in the GC participants that a greater anxiety trait was detected, whereas the Non-GC individuals showed no signs of the anxiety trait (P<75), i.e., the former group tended to perceive the situations as threatening, with intensification of the anxiety state. Thus, in the GC participants, the elevation of the anxiety state may be related not only to the fact that they already have an elevated trait but, as also observed in the Non-GC participants, but also it seems to be related to the current situation which includes their role as caregivers.

The concept of caregiver load covers important areas in the life of these individuals such as mental well-being, personal relations, physical overload, social support, finances and environment related to the caring of incapacitated adults.^9^ The results of the Caregiver Load Scale did not reveal a significant difference between GC and Non-GC participants. In addition, the scores were higher than those reported by Medeiros,^4^ indicating the presence of overload among these caregivers. Finally, analysis of the subscale of the Caregiver Load instrument revealed a correlation between the dimensions of general tension, disappointment and emotional involvement, indicating a subjective experience of overload when caring for persons with dementia.

Indeed, the individuals who participated in the GC had a higher educational level than those who did not participate. There were a larger number of persons holding jobs, even though they were acting in the capacity of housewives at the time. Although females predominated in both groups, there was a greater presence of males among GC participants.

Mean age was similar for the two groups, where children of patients predominated in the GC, whereas spouses predominated in the Non-GC. The greater participation of children of patients as caregivers in the GC seems to suggest a greater adhesion to this complementary therapeutic procedure, while the female presence may reflect the cultural roles attributed to women, especially regarding care for family members. Gonçalves et al.^8^ questioned elderly individuals about the person to whom they would resort for care in the event of disease and/or of limitations in self-care. The most frequent reply given by female respondents was that they would resort to their daughters, their spouses, granddaughters and sisters. The most frequent reply given by male respondents was that they would resort to their wife, indicating that women are more frequently associated with care, an observation repeated in the present study. Under the present conditions, a superposition of roles for females was noted, i.e., having a job and/or being a housewife while also being the caregiver for a patient with dementia.

GC participants also perceive themselves as the sole caregivers although they report relying on more caregiving help than Non-GC individuals, who believe they have no help. These data suggest that a higher educational level, greater social interaction outside the family context through job activities, a probably more extensive repertory of information and knowledge, as well as having auxiliary support for patient care, seem to be conditions associated with participation in this group.

At the same time, this modality of intervention has proved to be a considerably rich source of information for future studies, including assessments at the beginning of group formation and after 3 months of intervention,^14,20^ as well as the staging of dementia severity and etiology. Indeed, we should have more information on the history of the relationship between the caregiver and patient that might influence the present. Further, instruments that have been previously adapted to the Brazilian population should be preferred, as should those able to evaluate subjective burden of caregivers.

## Conclusion

Therefore, on the basis of the present study, we may conclude that although important psychosocial differences exist between the two groups, both are equally subjected to a significant psychological impact, state of anxiety and overload due to their roles as caregivers of persons with dementia.

We also conclude that the proposal of multidisciplinary non-medicamentous intervention in the model of Caregivers Groups seems to benefit participants, with probable positive repercussions on their patients, especially regarding patients’ quality of life.
